# APC mutation correlated with poor response of immunotherapy in colon cancer

**DOI:** 10.1186/s12876-023-02725-3

**Published:** 2023-03-28

**Authors:** Bing Li, Guoliang Zhang, Xuejie Xu

**Affiliations:** 1 Department of Medical Oncology, The Affiliated Hospital of Putian University, No. 999 Dongzhen Road, Licheng District, Putian, Fujian 351100 China; 2 Department of Thyroid Surgery, The Affiliated Hospital of Putian University, Fujian, 351100 China

**Keywords:** APC mutation, colon cancer, Tumor mutation burden, Immunotherapy, Immune cell infiltration

## Abstract

**Objective:**

APC (adenomatous polyposis coli) gene mutation is a central initialization in colon cancer tumorigenesis. However, the connection between APC gene mutation and immunotherapy efficacy for colon cancer remains unknown. This study aimed to explore the impact of APC mutation on immunotherapy efficacy for colon cancer.

**Methods:**

Colon cancer data from The Cancer Genome Atlas (TCGA) and Memorial Sloan Kettering Cancer Center (MSKCC) were used for the combined analysis. Survival analysis was performed to evaluate the association between APC mutation and immunotherapy efficacy in colon cancer patients. The expressions of immune check point molecules, tumor mutation burden (TMB), CpG methylation level, tumor purity (TP), microsatellite instability (MSI) status and tumor-infiltrating lymphocyte (TIL) in the two APC status were compared to evaluate the associations between APC mutation and immunotherapy efficacy indicators. Gene set enrichment analysis (GSEA) was performed to identify signaling pathways related to APC mutation.

**Results:**

APC was the most frequently mutated gene in colon cancer. The survival analysis demonstrated that APC mutation was correlated with a worse immunotherapy outcome. APC mutation was associated with lower TMB, lower expression of immune check point molecules (PD-1/PD-L1/PD-L2), higher TP, lower MSI-High proportion and less CD8 + T cells and follicular helper T cells infiltration. GSEA indicated that APC mutation up-regulated mismatch repair pathway, which may play a negative role in evoking an antitumor immune response.

**Conclusion:**

APC mutation is associated with worse immunotherapy outcome and inhibition of antitumor immunity. It can be used as a negative biomarker to predict immunotherapy response.

## Introduction

Colon cancer is one of the leading malignant tumors threatening human life. Worldwide, colon cancer is the third most frequent and the second most deadly of all cancers. It was reported that more than 25% of patients have been found metastasized when were diagnosed [1–3], suggesting a highly aggressive feature of colon cancer. The median 5-year survival rate of metastatic colon cancer remains poor, only 12.5% in the USA [3]. Conventional chemotherapy resistance and side effects owing to the toxicity are still the major challenges in the treatment of colon cancer. Hence a more effective and less toxic treatment for the disease is still needed.

Emerging evidences have shown that immunotherapy including programmed death-1 receptor s (PD1) and cytotoxic T-lymphocyte antigen 4 (CTLA-4) inhibitors has a repressive effect on a variety of tumors [4]. Nowadays immunotherapy has become an important option for the treatment of multiple cancers and made significant breakthroughs in treating colon cancer [5]. Some well-recognized molecular determinants such as TMB [6, 7], PD-L1 expression [8], DNA mismatch-repair deficiency [9–11] and TIL [12] have been identified associated with immunotherapeutic responsiveness. However, the efficacy of immunotherapy for advanced colon cancer remains unsatisfactory. A novel biomarker that can more accurately and effectively predict the efficacy of immunotherapy is still needed.

APC mutation, which results in an increased intestinal epithelial cell proliferation and loss of differentiation, has been proved playing a key role in the oncogenesis and progression of colon cancer [13, 14]. However, studies investigating the relationship between APC mutations in colon cancer and the efficiency of immunotherapy are still relatively lacking.

In this study, we explored TCGA database to get a landscape of somatic mutations for colon cancer. Then cBioPortal database was used to analyze the association between APC mutation and immunotherapy efficacy. APC mutations were analyzed for association with TMB, Immune checkpoint molecules expression, CpG methylation level, Tumor purity (TP), Microsatellite instability (MSI) status and tumor-infiltrating lymphocyte (TIL). TIL immune-related pathways were researched. The finding will provide a predictive value with APC mutation for immunotherapy response of colon cancer.

## Materials & methods

### Data acquisition

Somatic gene mutations data, gene transcription data, and corresponding clinical information of colon cancer cohort were downloaded from TCGA database (http://portal.gdc.cancer.gov/ projects). Clinical data of 85 colon cancer patients of MSKCC dataset were downloaded from cBioPortal database (https://www.cbioportal.org). All the enrolled patients should have full information including: gender, age, TMB, overall survival time, APC and survival status.

### Prognostic value assessment of APC mutation for the colon cancer cohort with immunotherapy

Colon cancer dataset from MSKCC were selected for the analysis. Survival analysis using Kaplan-Meier model was performed to assess the impact of APC mutation on overall survival status. Multivariate analysis using cox regression model was performed to assess the correlations between overall survival and clinical characteristics.

### Calculation of somatic gene mutation frequency in colon cancer sample

Somatic mutation data of colon cancer obtained from TCGA database was analyzed to acquire gene mutation frequency information. The top 30 mostly frequent mutated genes were displayed as oncoplot.

### Estimation of the correlation between APC mutation, CpG methylation, tumor purity, TMB and immune check point molecules

TMB refers to the number of somatic nonsynonymous mutations in the test genome region, usually expressed as mutations per megabase (MUT / Mb). TMB can indirectly reflect the ability and degree of neoantigen generation and predict the efficacy of immunotherapy for various tumors [15]. TMB was calculated using the TCGA somatic mutation data. CpG methylation data was obtained from UCSC Xena web (https://xenabrowser.net/datapages/). Tumor purity reflects the proportion of stromal cells and infiltrated immune cells in tumor microenvironment [16]. R software and “estimate” package were used to calculate tumor purity with colon cancer expression data from TCGA database. The expression of immune check point molecules involving PDCD1 (PD-1), CD274 (PD-L1) and PDCD1LG2 (PD-L2) were calculated with colon cancer transcription data. The mutation data, TMB, mean CpG methylation, Tumor purity, and transcription data were merged depending on the sample identity. Then these data was grouped by APC status and compared.

### Microsatellite instability prediction in colon cancer

Microsatellite instability (MSI) was predicted with a fifteen genes prediction module involving DDX27, EPM2AIP1, HENMT1, LYG1, MLH1, MSH4, NHLRC1, NOL4L, RNLS, RPL22L1, RTF2, SHROOM4, SMAP1, TTC30A, and ZSWIM3 in colon cancer transcription data in TCGA [17]. “PreMSIm” package of R was used for this analysis. Then MSI proportions in APC mutation type and wild type were compared.

### Immune cells infiltration analysis in colon cancer

In the bulk tumor sample, the 22 tumor-infiltrating lymphocyte (TIL) subset proportions was applied to assess the relative abundance of immune cell infiltration. CIBERSORT [18] was used to calculate the composition of TIL. Based on APC status, patients was classified as mutation and wild type. Then comparison of immune cell infiltration abundance was performed to evaluate the impact of APC mutation on immune cell infiltration in tumor sites in patients with colon cancer.

### Enrichment pathway analysis related to APC mutation

Gene set enrichment analysis (GSEA) [19] was performed using Broad Institute GSEA software (version 4.1.0) to identify signaling pathways related to APC mutation. TCGA colon cancer dataset was used for this analysis. Patients were divided into two groups according to APC status, and 1000 permutations was set as a normalized enrichment score (NES).

### Statistical analysis

The R software (version 4.0.2) was applied in Statistical analyses. Survival analysis was performed using Kaplan-Meier model, multivariate analysis using cox regression model was performed to assess the correlations between overall survival and clinical characteristics. The statistical significance was set as p-value < 0.05.

## Results

### The somatic mutations overview in colon cancer

**Fig. 1:**
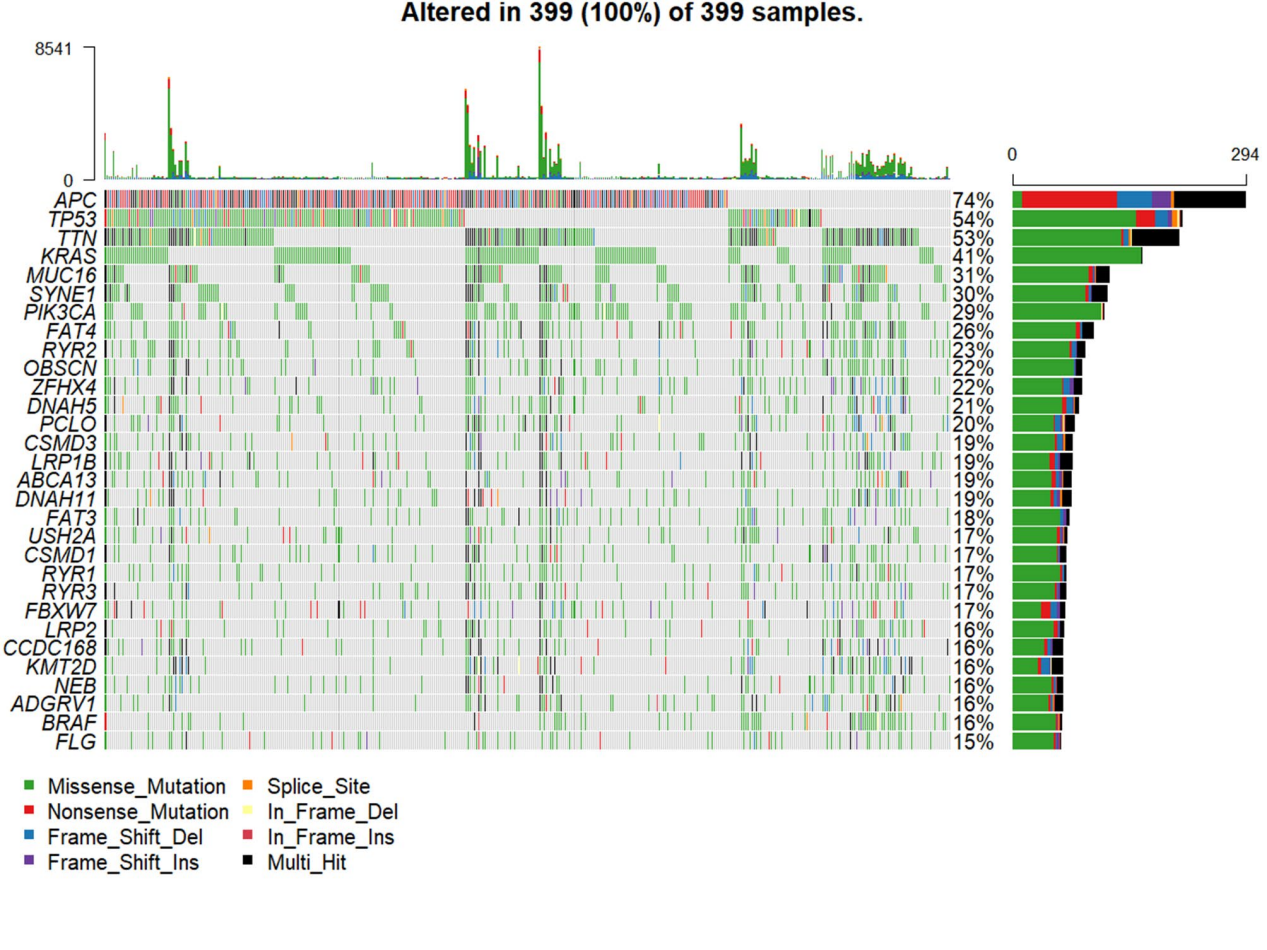
The somatic mutations overview in colon cancer. Dataset was obtained from TCGA database. The analysis displayed the top 30 most frequently mutant genes in colon cancer, and APC was the first one

Mutation frequencies of genes in colon cancer were analyzed using TCGA somatic mutation data. The top 30 mutated genes were classified as cancerous genes, of which the top 10 were APC (74%), TP53 (54%), TTN (53%), KRAS (41%), MUC16(31%), SYNE1(30%), PIK3CA (29%), FAT4(26%), RYR2(23%), OBSCN (22%) (Fig. 1). APC is the most frequently mutated gene in colon cancer, and it is also an important gene in the occurrence and development of colon cancer. MSKCC data showed that there were no significant differences in gender, age, TMB, tumor purity, primary site and type of drug between the APC mutant group and the wild group (Table 1).

**Table 1:** Clinical characteristics of patients in MSKCC dataset

	APC			
	Mutation type (n = 60)	Wild type (n = 25)	t/χ^2^	P-value
Sex(male/female)	35/25	14/11	< 0.01	1.000
Age	53.4 ± 12.1	58.4 ± 12.8	1.66	0.104
TMB	26.4 ± 42.7	37.3 ± 34.0	1.25	0.217
Tumor purity	39.0 ± 17.7	37.9 ± 22.6	-0.20	0.841
Primary site			5.69	0.320
Cecum	8	6		
Ascending colon	9	3		
Transverse colon	8	4		
Descending colon	4	1		
Sigmoid	22	4		
Unclassified	9	7		
Therapeutic regimen			0.52	1.000
PD-1/PD-L1	53	23		
CTLA4	1	0		
Combo	6	2		

TMB: tumor mutation burden; CTLA4: cytotoxic T lymphocyte-associated antigen-4; PD-1: programmed cell death protein 1; PD-L1: programmed cell death 1 ligand 1. P<0.05 is considered statistically significant

### APC mutation and survival prognosis

**Fig. 2:**
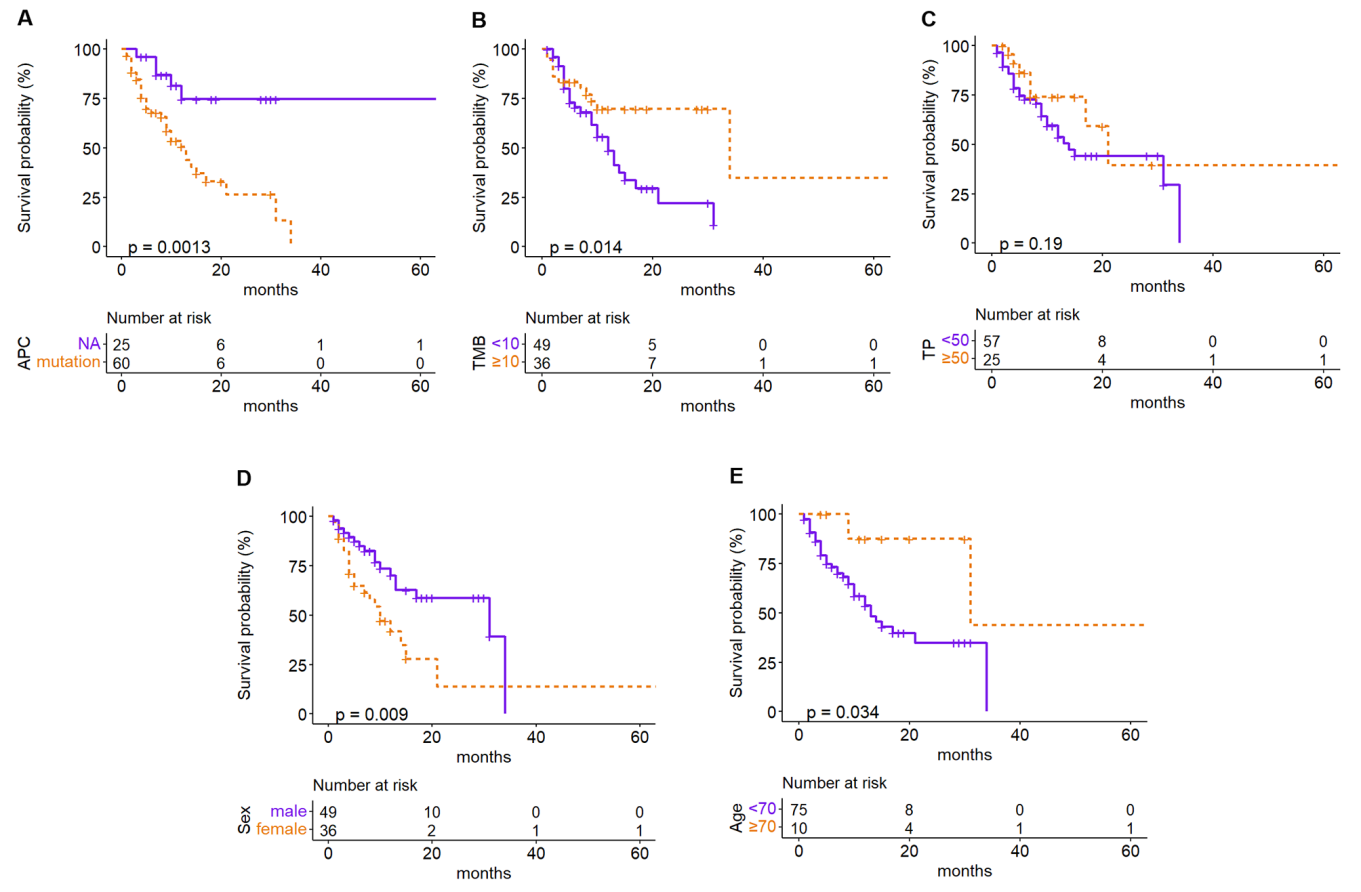
Survival analysis for colon cancer was performed using Kaplan-Meier model. (**A**-**E**) Overall survival analysis based on different characteristics of colon cancer immunotherapy cohort

**Fig. 3:**
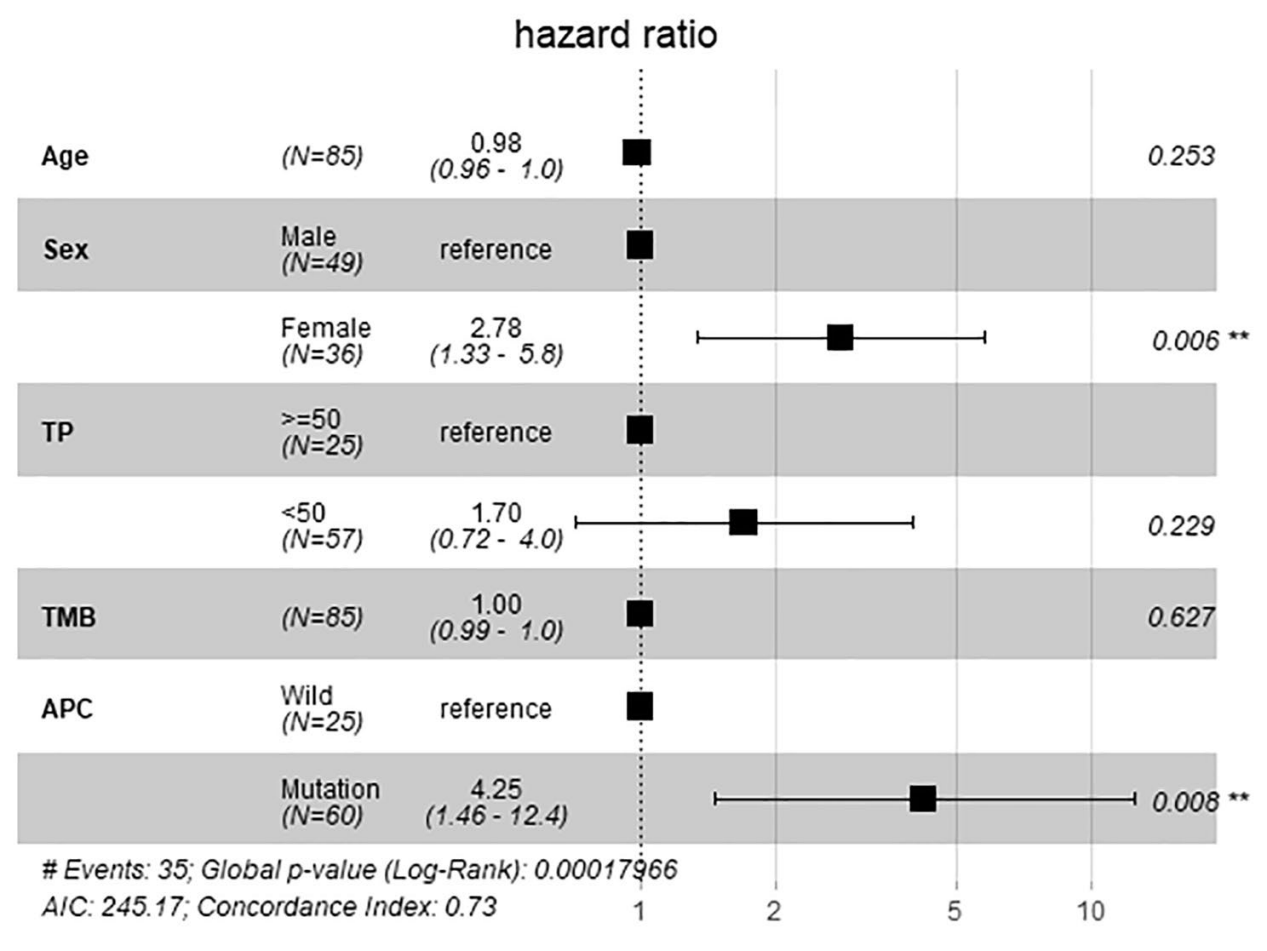
Multivariate analysis of factors influencing overall survival using Cox’s regression model. HR: Hazard ratio; CI: Confidence interval; P<0.05 was considered with statistically significant difference

The Kaplan-Meier survival analysis was used to analyze the link between APC gene status and immunotherapy outcomes in colon cancer patients. The analysis suggested that the group of patients with APC mutation had a much worse response for immunotherapy than the wild type group (Fig. 2). Then Cox’s proportional hazard model was adopted to determine the independent risk factors and the result was further validated (Fig. 3). In the multivariate analysis, APC mutation (HR = 4.25; 95% CI, 1.46–12.4; p = 0.008) and female (HR = 2.78, 95% CI, 1.33–5.8; p = 0.006) were associated with worse immunotherapy response. It suggested that APC mutation could be one of the main factors that impacted the immunotherapy efficacy of colon cancer patients.

### APC mutation associated with TMB, TP and immune check point molecules expression

**Fig. 4:**
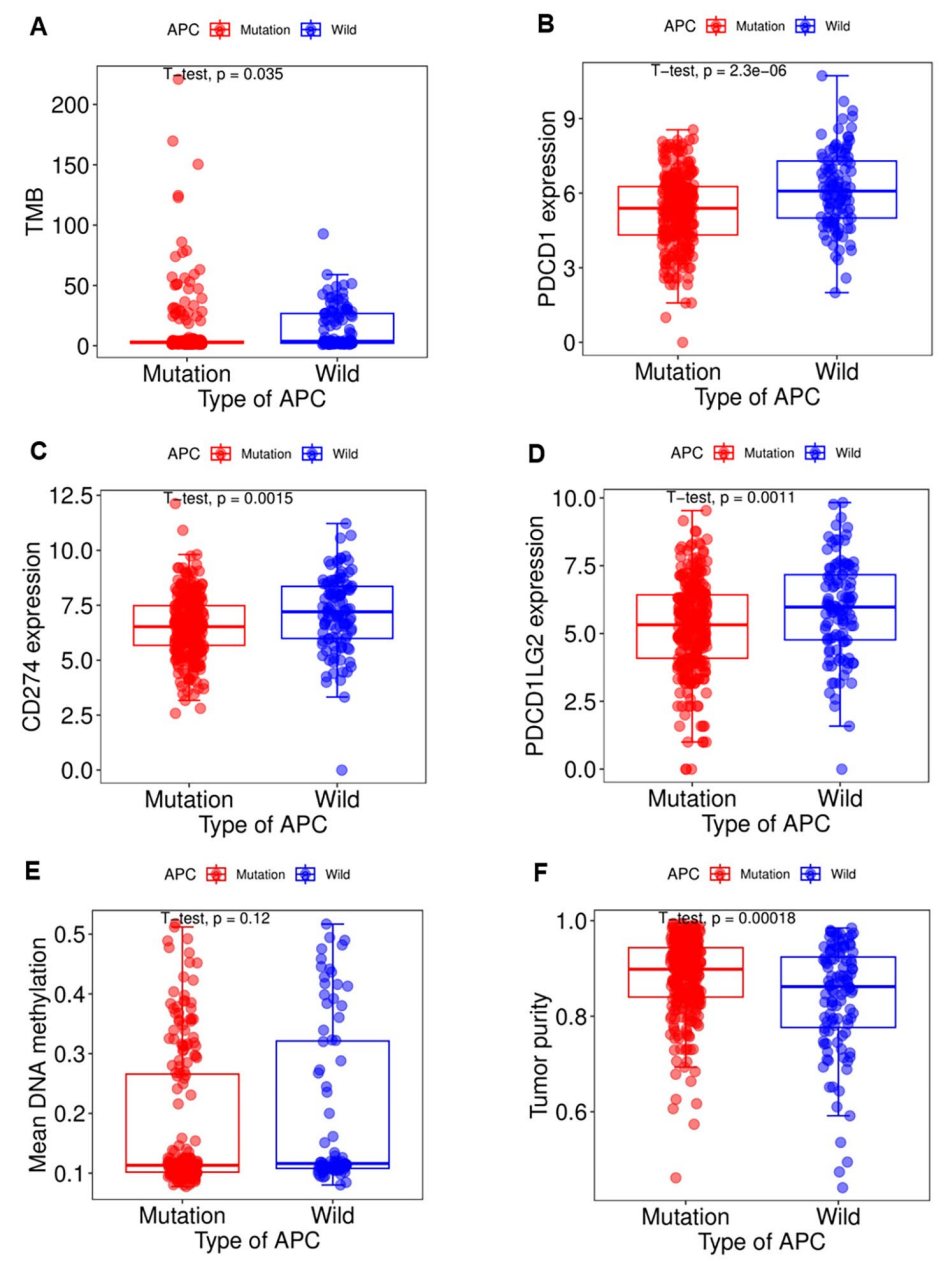
The association between APC mutation and TMB, immune checkpoint molecules expression, mean CpG methylation and tumor purity. (**A**) APC mutation was associated with a lower TMB. (**B**-**D**) APC mutation was correlated with lower expression of PDCD1 (PD-1), CD274 (PD-L1) and PDCD1LG2 (PD-L2). (**E**) APC status was not associated with CpG methylation level. (**F**) APC mutation was associated with higher tumor purity

High TMB [6, 7] and PDL1 [8] expression were considered as positive biomarker of immunotherapy efficacy. Some studies reported that PD-L2 had a similar effect as PD-L1, which interacted with PD-1 suppressing T-cell proliferation, cytokine production, and T-cell cytolysis [20–22].Therefore, the relationship between APC gene mutation and TMB/immune checkpoint molecules expression was analyzed. Analysis of colon cancer cohort in TCGA database showed that APC mutation was associated with a lower TMB and was associated with a lower genes expression of PDCD1 (PD-1), CD274 (PD-L1) and PDCD1LG2 (PD-L2) (Fig. 4A-F).

### APC mutation correlated with a lower rate of MSI-High in Colon cancer

**Fig. 5:**
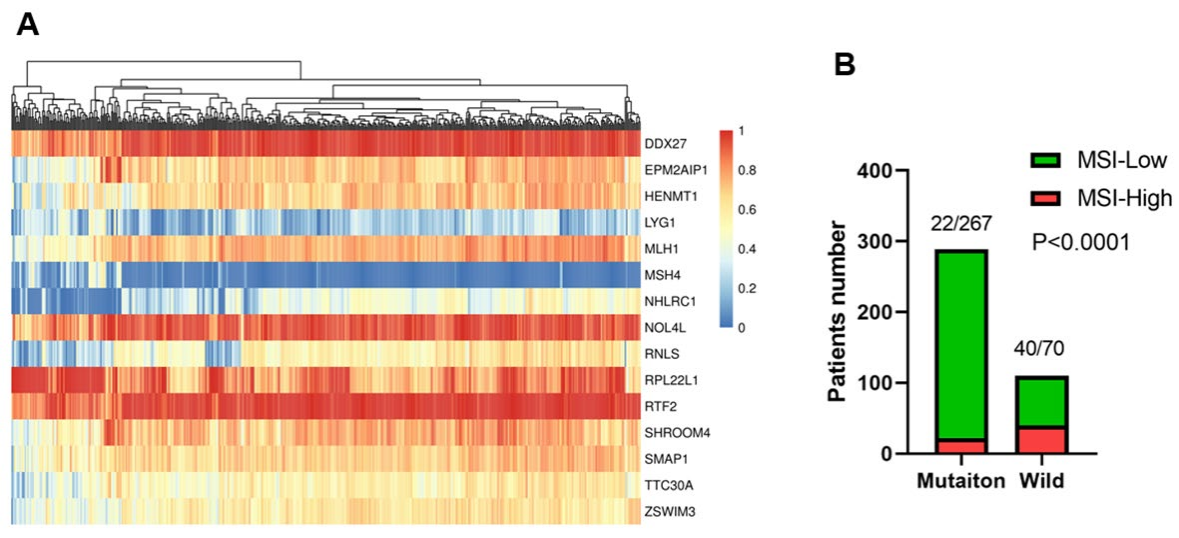
The association between APC mutation and MSI-H status (**A**) Heatmap of fifteen genes module with 0–1 scaled expression for the calculation of MSI in TCGA colon cancer cohort. (**B**) The proportion of MSI-H in patients with different APC status. P<0.05 was considered with statistically significant difference

MSI-High was used as a predictive biomarker for the clinical application of immunotherapies [23]. We calculated the expression with 0–1 scale for the fifteen genes in each sample (Fig. 5A).Then MSI status were predicted for colon cancer cohort from TCGA. The results suggested that patients with APC gene mutation showed a significant lower rate of MSI-High compared with patients with APC wild type (P < 0.001, Fig. 5B).

### The immune cell infiltration correlated with APC mutation in colon cancer

**Fig. 6:**
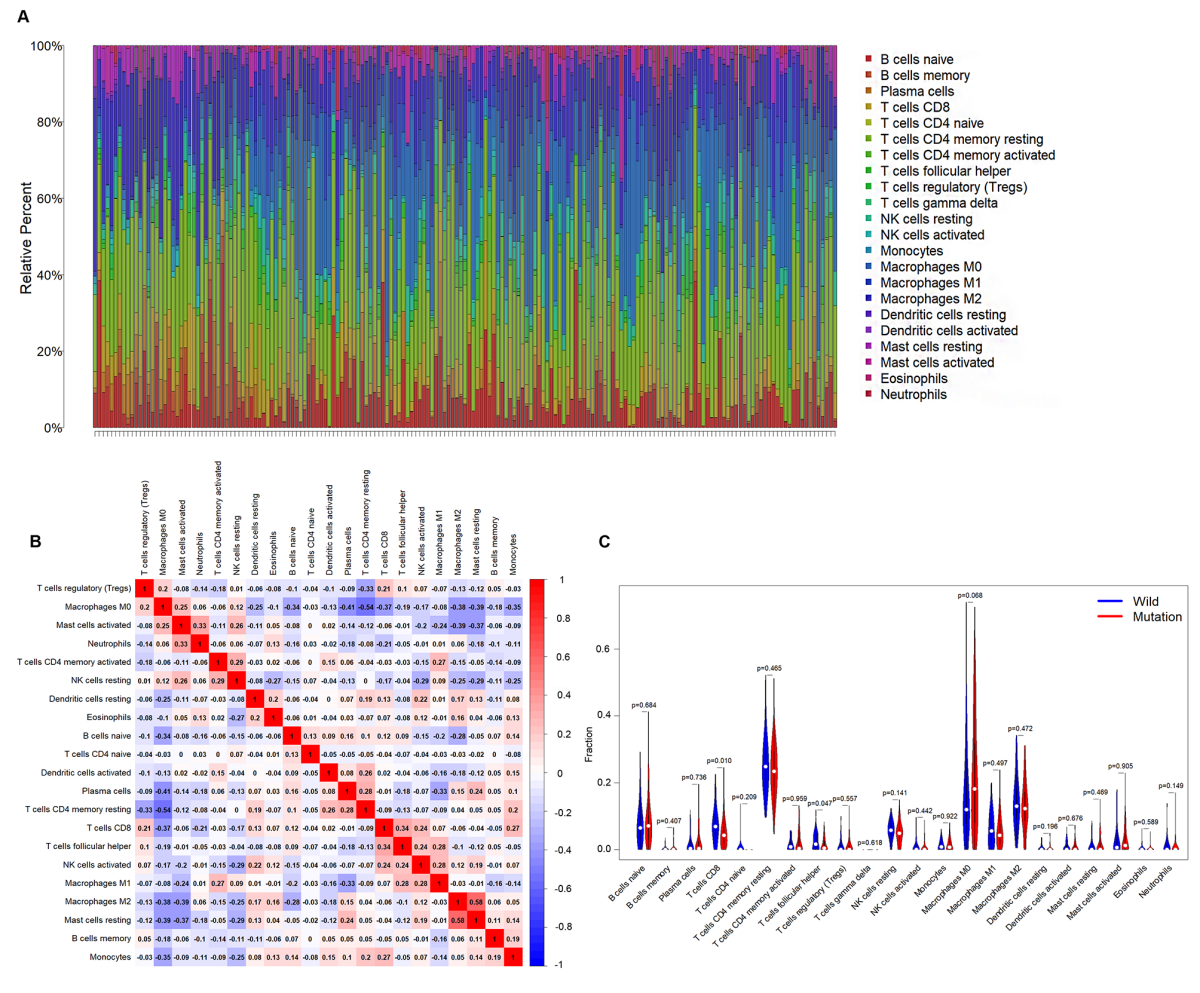
The immune cell infiltration correlated with APC mutation in colon cancer. (**A**) Distribution of 22 immune cells infiltrated in every specimen with bar chart. (**B**) Thermal map of correlation coefficient of immune cell. The redder the color, the greater the positive correlation while the bluer the color, the greater the negative correlation. (**C**) The differences in immune cells between APC-mutant group and APC-wild group with violin plot

The CIBERSORT analysis revealed significant differences in the composition of the 22 types of immune cells in each sample (Fig. 6A). Immune cell correlation coefficient heat map showed that M0 macrophages were moderately negatively correlated with quiescent CD4 + memory T cells (R= -0.54) and plasma cells (R=-0.41). M2 macrophages were moderately positively correlated with quiescent mast cells (R = 0.58) (Fig. 6B). In addition, we found that CD8 + T cells and follicular helper T cells, which play an important role in antitumor immunity, were significantly less enriched in patients with APC mutations, while other immune cells were not significantly different between the two groups (Fig. 6C).

### Enrichment pathway analysis of APC mutation

**Fig. 7:**
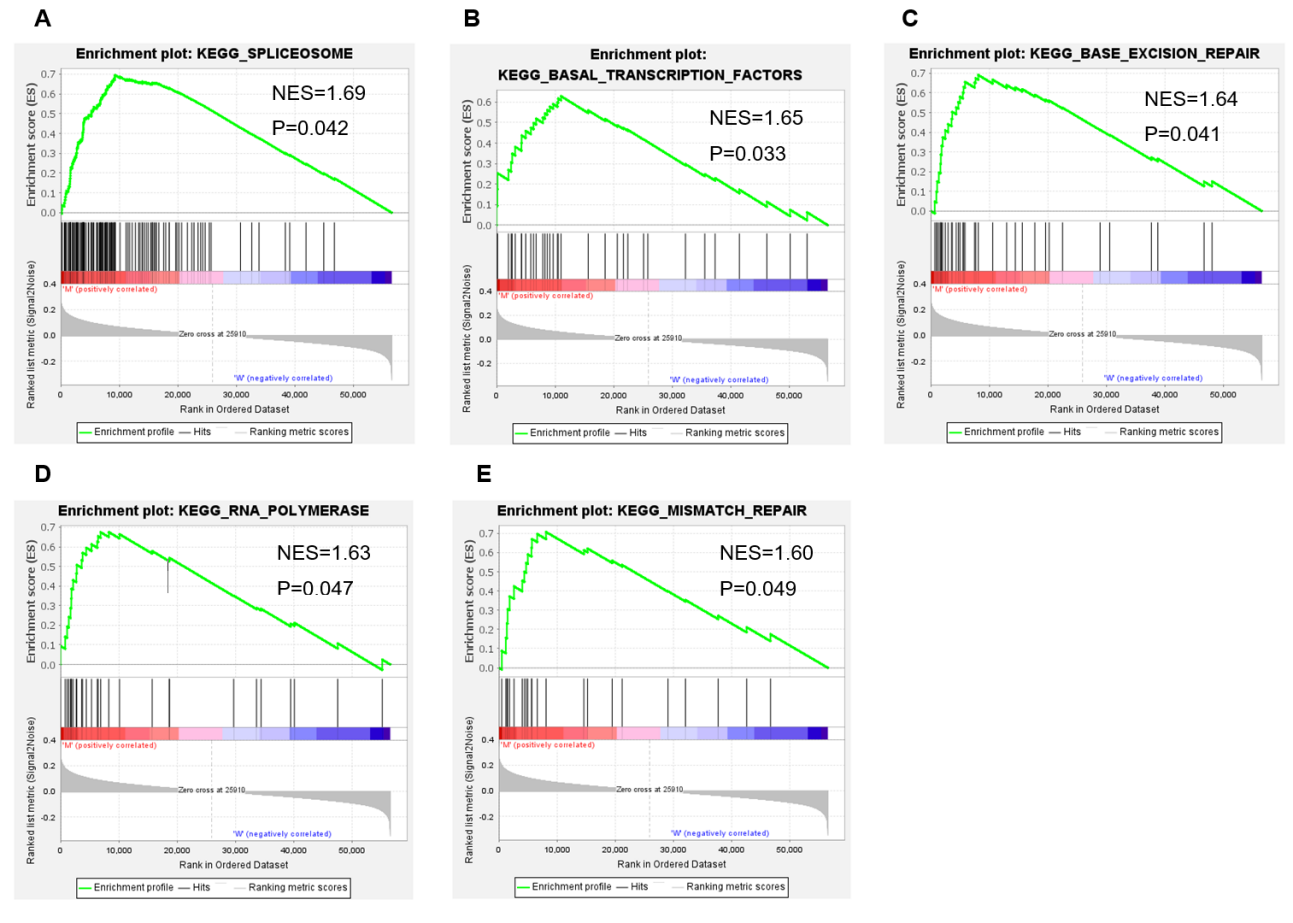
APC mutation related pathway enrichment. (**A**) Spliceosome Pathway, (**B**) Basal transcription factors Pathway, (**C**) base excision repair Pathway, (**D**) RNA polymerase pathway, and (**E**) Mismatch repair pathway is enriched in APC-mutant group. P < 0.05 was considered with statistically significant difference

GSEA performed with TCGA colon cancer data revealed that Spliceosome Pathway, Basal transcription factors Pathway, Base excision repair, RNA polymerase, and Mismatch repair pathway were significantly enriched in APC-mutant group (Fig. 7A-E).

## Discussion

Currently, immunotherapy is more and more widely used in cancer therapy. The studies of efficacy and predictive indicators of immunotherapy have become a research hotspot in the field of cancer. It is well known that APC gene mutations are responsible for familial adenomatous polyposis (FAP), and also play an important role in the initiation and development of colon cancer [24–26]. In this study, we discovered that APC was closely correlated with poor response of immunotherapy in colon cancer. Moreover, in further data analysis, we found that APC mutations were strongly associated with lower TMB and lower expression of immune checkpoint molecules like PD-1, PD-L1 and PD-L2. We have noted a contradiction in our analysis of the MSKCC dataset and TCGA database. To address this issue, we conducted a thorough review of the methodology and limitations of each dataset. We found that differences in patient populations, sample sizes, and methods of data collection and analysis between the MSKCC dataset and TCGA database could have contributed to the contradictory results. Thus, we acknowledge the limitations of using different datasets in our analysis and interpret our findings. We will utilize larger and more homogeneous datasets in future studies to elucidate the relationship between APC mutation and TMB. Our study of colon cancer patients treated with immunotherapy from the cBioPortal database suggested that patients with APC mutations had a much worse overall survival rate than that with APC wild type.

PD-L1 and PD-L2 are the two ligands of PD-1 receptor. The interaction of the PD-1 receptor on T cells with its ligands has a negative impact on T-cell–mediated immune responses and leads to immune escape in human tumors [27]. Previous studies have reported that patients with a higher PD-L1 expression level have a better immunotherapy response, especially with PD-1/PD-L1 blockade, and PD-L1 expression was considered as a predictive biomarker of immunotherapy in diverse cancers [12, 28–33]. The interaction between PD-1 and PD-L2 can generate similar effects, such as inhibiting T-cell proliferation, cytokine production, and T-cell cytolysis [20, 21]. Yearley et al. [22] reported that PD-L2 positivity was a significant predictor of PFS in head and neck squamous cell carcinoma (HNSCC) and also associated with OS in HNSCC. It was believed that high TMB can stimulate more neoantigens that may enhance the response to immune checkpoint inhibitors^6^. TMB has been shown to have a close correlation with response to PD-1/PD-L1 blockade in multiple cancers [10, 11, 34–37]. Previous studies have demonstrated that patients with a lower TMB and PD-L1 expression had significantly lower response rates, and shorter PFS and OS than those with a higher one [38]. In this study, we discovered that patients with APC mutation had a much worse overall survival rate than that with APC wild type. Cox’s multivariate analysis suggested APC mutation was an independent risk factor of prognosis in colon cancer patients treated with immunotherapy. APC mutation was negatively correlated with TMB, PD-1, PD-L1 and PD-L2 expression, which might be one of the reasons that colon cancer patients can’t benefit from immunotherapy.

In recent years, increasing evidences suggested that the tumor immune microenvironment (TIM) played a significant role in predicting prognosis and evaluating therapeutic efficacy [39–41]. Previous studies have reported that TIL density associated with up-regulated PD-L1 and clinical results. CD8 + TIL is considered to be a vital executor in tumor-killing immunity and maintain immunization surveillance [42–47], and follicular helper T cells facilitate the anti-tumor response in the immune checkpoint therapy [48]. Rich infiltration of CD8 + tumor-infiltrating lymphocytes, CD4 + T helper cells and activation of type I interferon signaling were associated with the signature of dMMR-MSI colon cancer, which was considered to benefit from immunotherapy [49]. In this study, we observed that APC mutation was associated with decreased CD8 + T cells and follicular helper T cell infiltrations. Thus, we concluded that APC mutation may mediate colon cancer resistance to immunotherapies by reduce CD8 + T cells infiltration and immune checkpoint gene expression. The mechanisms need further studies.

Mismatch repair pathway was functioned as identifying and repairing mismatched bases in the process of DNA replication and genetic recombination in normal and tumor cells. The defection of the proteins of this pathway, which was called mismatch repair deficiency (dMMR), will lead to microsatellite instability-high (MSI-H) and result in the accumulation of TMB in cancer-related genes and the generation of neoantigens, thereby stimulates the anti-tumor immune response ultimately [23]. In clinical work, dMMR predicts a good response of immune checkpoint blockade therapies, and was considered as a biomarker for immunotherapies [50]. In this study, gene set enrichment analysis showed a significant enrichment of mismatch repair pathway in APC-mutant colon cancer. As a result, enriched mismatch repair pathway may play a negative role in evoking an antitumor immune response conversely.

The major limitation of this study is that the size of colon cancer cohort with immunotherapy downloaded from cBioPortal database was small, only 85 patients were involved. The mechanisms that APC mutation affecting immune checkpoint gene expression and mismatch repair pathway are not clear. Further studies are needed to explore the specific molecular mechanisms.

## Conclusion

In conclusion, we demonstrated that the colon cancer patients with APC mutations were associated with poor response of immunotherapy. APC mutation can be served as a negative biomarker for colon cancer immunotherapy.

## Data Availability

The datasets used and/or analyzed during the current study are available from the corresponding author on reasonable request.
